# The Direct and Indirect Relationship Between Social Cognition and Psychosocial Dysfunction in Major Depressive Disorder

**DOI:** 10.3389/fpsyt.2019.00347

**Published:** 2019-05-17

**Authors:** Matthew J. Knight, Bernhard T. Baune

**Affiliations:** ^1^Discipline of Psychiatry, Adelaide Medical School, University of Adelaide, Adelaide, Australia; ^2^Department of Psychiatry and Psychotherapy, University Hospital Münster, University of Münster, Münster, Germany

**Keywords:** depression, social cognition, cognition, mediation, memory, facial recognition, psychosocial functioning

## Abstract

**Background:** Recent evidence suggests that depressed patients experience social cognitive deficits (e.g., poor affect recognition). However, very little is known regarding the contribution of social cognitive deficits to psychosocial dysfunction (e.g., occupational functioning). In particular, the mechanistic roles of depression severity and cognitive deficits (e.g., memory) in this domain have not been explored. The current study evaluated the extent to which mood symptoms and cognitive deficits provide a mechanistic explanation for the relationship between social cognitive and psychosocial deficits in major depressive disorder (MDD).

**Methods:** Data were obtained from 111 participants with MDD (75 Female, mean age = 35, 84% Caucasian, 12% Asian, 4% Other) in the Cognitive Function and Mood Study (CoFaM-S), a cross-sectional study of mood, social cognition, cognition, and psychosocial functioning in mood disorders. Social cognitive abilities were assessed using the Social Perception subtest of the Wechsler Adult Intelligence Scale, and psychosocial dysfunction was clinically evaluated with the Functioning Assessment Short Test (FAST).

**Results:** Cognitive deficits and mood symptoms did not significantly mediate relationships between social cognitive ability and psychosocial dysfunction. The exception was executive function, which mediated an indirect relationship between meaning interpretation (i.e., theory of mind) and self-perceived cognitive dysfunction.

**Conclusion:** The results suggest that the relationship between social cognitive deficits and psychosocial dysfunction is not mechanistically explained by mood symptoms or nonsocial cognition. Development of treatment strategies targeting social cognitive deficits in patients with MDD is warranted.

## Introduction

“Social cognition” refers to the broad range of perceptual and cognitive abilities involved in the interpretation of social information, including affect recognition, eye contact, prosody interpretation, and inferring the mental states of others (i.e., theory of mind) (1, 2). Recent evidence suggests that major depressive disorder (MDD) is associated with social cognitive deficits in both the acute (3) and remitted stage of illness (4), primarily in domains of affect recognition and theory of mind (5–8). However, the extent to which social cognitive deficits independently contribute to psychosocial dysfunction (e.g., occupational functioning, interpersonal relationships) has not been explored.

Social cognition is reliant on a number of cognitive abilities, as social information is processed through memory and attention, with more complex social abilities (e.g., theory of mind) likely facilitated by executive functions (e.g., problem solving) (9, 10). Evidence for the reliance of social abilities on cognitive function in mentally ill patients is available in domains of schizophrenia and attention deficit hyperactivity disorder (ADHD), with research demonstrating that deficits in processing speed, executive functioning, and working memory are associated with impaired theory of mind (1, 9, 11, 12). These findings highlight the close overlap between social and nonsocial cognition in clinical populations, and the need for research in the domain of MDD specifically.

A wide body of literature supports the notion that cognitive deficits are associated with psychosocial dysfunction, notably in domains of occupational functioning, interpersonal relationships, daily responsibilities, and self-perceived quality of life (13–18). Cognitive deficits acquired in the acute stage of illness commonly persist through diagnostic remission and are associated with higher rates of relapse and prolonged psychosocial dysfunction (19–21), highlighting the chronicity of dysfunction associated with cognitive deficits. Given the broad links between cognitive and psychosocial dysfunction, it is possible that social cognitive deficits also contribute to functional deficits in MDD (8, 22), particularly in domains highly reliant on social functioning (e.g., occupational functioning, interpersonal relationships).

An initial study by Knight and Baune (22) demonstrated that prosody interpretation was linked with reduced psychosocial function, whereas facial affect recognition and meaning interpretation (e.g., sarcasm detection) were not associated with functional deficits. These findings highlight the clinical importance of social cognition in MDD patients, such that remediating social cognitive deficits may be an important treatment target in alleviating psychosocial dysfunction. At present, there is a dearth of literature examining the role of social cognition in psychosocial function, highlighting the need for studies that show the impact and clinical mechanisms of social cognition in MDD.

As social cognition is likely reliant on cognitive abilities (e.g., memory), the question is raised as to whether social cognition contributes to psychosocial functioning independently of cognitive functioning. It is possible, for instance, that deficits in executive functioning could explain impaired analysis of social information and reduced psychosocial functioning. If executive function is the primary clinical mechanism, then patients presenting with social cognitive deficits may benefit more from treatment targeting executive function specifically than from treatments centered on social cognition. Given the importance of selecting appropriate treatment targets, it is crucial to elucidate whether cognitive domains mediate the relationship between social cognition and psychosocial function. The current study is the first to evaluate the extent to which the effect of social cognition on psychosocial function is mediated by cognitive performance.

Social cognitive deficits in MDD are also associated with the general severity of illness symptoms (e.g., pessimism, anxiety) (5, 23, 24), suggesting that general depression symptoms interfere with the perception and interpretation of social information. Support for this notion was found by Air et al. (5), who found that greater affective symptom severity in currently depressed patients was negatively associated with ability to detect emotions and interpret meaning from verbal prosody. In addition, somatic symptoms (e.g., headaches, muscle pain) were negatively associated with ability to detect emotions from facial affect. The role of symptom severity in social cognition was also demonstrated by Gollan et al. (23), who presented MDD patients and healthy controls with pictures of facial expressions, which varied in the intensity of emotion conveyed. The results indicated that depressed patients detected sadness with greater sensitivity, but detection of other emotions (e.g., surprise) was impaired relative to healthy controls. Symptom severity enhanced this relationship, such that more severely depressed patients were increasingly sensitive to sadness, and less sensitive to other emotions. Taken together, available research suggests that greater depression severity may reduce social perception abilities in general (5), as well as facilitate hypersensitivity to negative social information (25).

Similarly to cognition, greater depressive symptom severity is associated with reduced social cognitive abilities and poor psychosocial functioning (14, 26). Accordingly, it is important to distinguish the contribution of social cognition to psychosocial functioning independently of illness severity, a key objective in the current study. This research will better inform clinicians as to the importance of treating social cognitive deficits as distinct from mood symptoms (27), or alternatively, whether treatment of mood symptoms by existing treatment approaches (e.g., cognitive behavioral therapy, antidepressants) is sufficient to remediate social cognitive deficits.

It is also possible that poor social cognition exerts a negative effect on psychosocial function indirectly by increasing symptom severity or cognitive dysfunction. These indirect effects would not have been detected by previous studies, which examined total (i.e., unmediated) relationships between social cognition and psychosocial function (22, 28). As a result, the present study fills an important gap in our understanding by examining the indirect relationship between social cognition and psychosocial function, as mediated by cognition and mood symptoms.

The primary goals were as follows:

1. To evaluate whether social cognitive domains (i.e., affect recognition, prosody interpretation, meaning interpretation) were directly associated with overall psychosocial dysfunction, independently of mood symptoms or cognitive deficits.
2. To examine whether social cognitive domains were associated with psychosocial dysfunction indirectly *via* mediating mood symptoms or cognitive deficits.

## Methods

### Participants

The present data were obtained from the Cognitive Function and Mood Study (CoFaM-S) (19), a multisite cross-sectional analysis of cognitive, social, emotional, and functional status in patients with mood disorders. The CoFaM-S was reviewed by the Human Research Ethics Councils (HRECs) of the Royal Adelaide Hospital (approval number: 111230) and the University of Adelaide (approval number: H-160-2011) and was conducted in accordance with the Declaration of Helsinki. All participants were verbally informed regarding study procedures, and read a study information sheet, before providing written consent to participate. CoFaM-S participants were at least 18 years of age, and no age limit was imposed. Inclusion criteria included lifetime diagnosis of a mood disorder according to *diagnostic and statistical manual of mental disorders, fourth edition, text revised (DSM-IV-TR)* criteria (29). Participants were recruited from inpatient and outpatient clinics throughout Eastern, Western, and Northern Mental Health Networks in Adelaide, South Australia, as well as through advertisement in the general community. Exclusion criteria included diagnosis of psychotic disorders, dementia, learning disorders, eating disorders, autism spectrum disorder, or medical illnesses, which could affect cognitive functioning (e.g., multiple sclerosis) (19). The mini international neuropsychiatric interview (MINI) Neuropsychiatric Diagnostic Interview was used to screen patients for psychiatric illness (30), and the Hamilton Depression Rating Scale (HAM-D) was used to measure depression symptom severity (31).

Participants (*N* = 111) with a lifetime diagnosis of MDD were included in the current study on the basis of completing standard measures of social cognition, cognition, depression symptom severity, and psychosocial functioning (see specific scales below). Inclusion criteria were a current (*n* = 42) or previous (*n* = 69) diagnosis of MDD following *DSM-IV-TR* criteria (29) according to the MINI 600 (31). Average time since first depressive episode was 15.2 years (SD = 14.04), and mean number of previous depressive episodes was 1.4 (SD = 1.39). Antidepressants were being used by 29 (26%) of the included participants. Participants’ mean age was 34.95 (SD = 16.38), 75 (68%) participants were female, and mean years of education were 13.48 (SD = 2.24). Ninety-three (84%) participants were Caucasian, 13 (12%) were Asian, and 5 (4%) were Indigenous/African/Hispanic.

### Social Cognitive Assessment

Social cognition was evaluated with the Wechsler Adult Intelligence Scale Advanced Clinical Solutions (ACS) Social Perception Subtest (WAIS-ACS). The assessment involves the detection of emotional states and meaning interpretation by appraisal of facial affect, prosody, and body language (32, 33). A previous study by Air et al. (5) indicated that social cognitive abilities measured by the ACS were associated with mood and anxiety symptoms in currently depressed, but not remitted, individuals. The findings of Air et al. (5) suggest that social cognition as measured by the ACS is a state-like feature of illness affected by patients’ present diagnostic status. Accordingly, the scale is appropriate for the current purpose of testing associations between social cognition and psychosocial function. The ACS assessment tasks are explained below:

- *Affect Naming (ACS Affect):* The participant is presented with 24 images of male and female faces, each displaying a particular emotion (i.e., happiness, sadness, surprise, fear, anger, disgust, and neutral). An answer sheet is provided to participants, indicating each of the seven potential responses (i.e., emotions) that could be displayed by each face image. Participants are instructed to select which emotion on the answer sheet best matches that conveyed by the image. Correct answers award a single point, leading to a maximum possible raw score of 24, demonstrating ability to detect facial affect.
- *Prosody–Face Matching (ACS Prosody):* Participants are presented with six faces, each displaying a particular emotion (i.e., the seven emotions used in the Affect Naming task). A voice is played to the participant, who must match the emotional tone of the speaker with one of the six presented faces. This process is repeated 12 times, for a maximum possible raw score of 12. This task reflects the ability to interpret prosody in the context of associated facial affect.
- *Prosody–Pair Matching (ACS Pairs):* In this task, the participant is presented with four images, each depicting two people interacting. Similar to the face matching task, a voice is played to the participant, who must indicate which image best matches the tone and intention of the speaker. In addition, the participant is instructed to indicate what feeling is conveyed by the tone of voice, and whether the tone of voice changes the meaning of the comment (e.g., sarcasm, humor). If a meaning change does occur, the participant indicates the true meaning/intentions of the speaker. Taken together, performance in the prosody–pair matching task leads to a maximum raw score of 42, indicating ability to interpret meaning from prosody and body language in social interactions.

### Cognitive Assessment

Cognitive functioning was assessed with three cognitive test batteries: the Repeat Battery for the Assessment of Neuropsychological Status (RBANS), the Colorado Assessment Tests (CATS), and the Psychology Experiment Building Language (PEBL). The RBANS is a brief screening instrument designed to detect neuropsychological deficits in individuals with physical or mental illness (34). Five cognitive domains are assessed: immediate memory, delayed memory, visuospatial/constructional memory, language, and attention. RBANS tests included in the current study were the digit span, list learning, list recognition, store memory, figure recall, picture naming, figure copy, and line orientation tests (34). The CATS battery included the *n*-back, visual span, word recall, Tower of London, and Wisconsin card sorting tests (35). The PEBL battery includes computerized versions of many psychological tests (e.g., the Tower of London, Corsi Blocks) (36). We utilized the Stroop and Wisconsin Card Sorting tasks in the PEBL battery as additional measures of executive functioning and attention. Individual participants completed either the CATS or PEBL executive tasks (i.e., Tower of London, Wisconsin Card Sorting test), as each test battery was applied at a different site of the study.

Domain-specific cognition composites were derived from the RBANS, CATS, and PEBL batteries by transforming raw performance in specific tasks (e.g., digit span, list learning) into *Z*-scores, which were averaged into composite *Z*-scores (e.g., immediate memory), giving equal weighting to each test from which the composite cognitive domain *Z*-score was derived. Individual participants completed either the CATS or PEBL executive tasks, as these test batteries were applied at different study sites. **Table 1** demonstrates the derivation test results to composite *Z*-scores in domains of executive functioning, attention, immediate memory, delayed memory, semantic fluency, and spatial cognition. For a similar procedure, see Godard et al. (37).

**Table 1:** Calculation of composite domain-specific *Z*-scores by cognitive domain in the Repeat Battery for the Assessment of Neuropsychological Status (RBANS), Colorado Assessment Tests (CATS), and Psychology Experiment Building Language (PEBL) batteries.

Cognitive domain	Cognitive tests		
	RBANS	CATS	PEBL
Executive Functioning		Tower of London (excess moves total) Wisconsin Card Sorting test (perseverative responses)	Stroop (incongruency errors) Tower of London (total excess moves) Wisconsin Card Sorting test (perseverative responses)
Attention	Digit Span Coding	VSPAN^†^ (total average time) NBACK (accuracy)	Stroop (incongruency errors)
Immediate Memory	List Learning Store Memory Digit Span	Word Recall (total words recalled) VSPAN (correct trials forward) VSPAN (correct trials backward)	
Delayed Memory	List Recognition List Recall Story Recall Figure Recall	Word Recall (primacy total)	
Semantic Fluency	Picture Naming Semantic Fluency	Word Recall (total words recalled)	
Spatial Cognition	Figure Copy Line Orientation	Tower of London (percentage above optimal)	

^†^VSPAN, verbal memory span.

It is noteworthy that several of the cognitive tests employed overlap across more than one cognitive domain. However, this should not preclude the current domain-specific use of these tests, as each is primarily associated with a specific domain. For instance, despite being an “executive” task, Beck’s Card Sorting Test (BCST) places significant load on visuospatial cognition due to its reliance on analyzing color, shape, and number of visual features. As a result, the BCST is not a “pure” measure of executive function. However, the primary cognitive challenge of the BCST is to adapt to new logical rules in sorting cards. In contrast, analysis of the visual information alone (i.e., identifying color/shape/number features) is relatively simple. As a result, we contend that it is appropriate to consider the BCST as a measure of executive function, as executive demands comprise the primary cognitive challenge of the task. Similarly, other cognitive tests also overlap several cognitive domains, but the most parsimonious explanation is to consider the primary cognitive domain of a particular cognitive test.

### Clinical Assessments

The Functioning Assessment Short Test (FAST) was used to clinically evaluate psychosocial functioning. The FAST is a clinician rated interview, in which the clinician conducts a semistructured interview to evaluate the extent of dysfunction across six functional domains (i.e., autonomy, occupational functioning, cognitive functioning, financial issues, leisure time, and interpersonal relationships). Functional impairment is rated on a four-point scale, with 0 indicating no dysfunction and 3 indicating severe impairment. Six composite FAST scores were derived from the mean score of impairment in each functional subdomain [i.e., autonomy (range: 0–12), occupational dysfunction (range: 0–15), subjective cognition (range: 0–15), financial issues (range: 0–6), interpersonal relationships (range: 0–18), and leisure time (range: 0–6)]. Overall psychosocial functioning (i.e., FAST total score) is indicated by the sum of FAST subdomains (range: 0–72). FAST total and FAST subdomain scores were used as the dependent variable in regression analyses.

Depression symptom severity was evaluated with the Hamilton Depression Rating Scale (HAM-D) (31). The scale involves 17 interview items that measure a range of mood and somatic symptoms, ranging in severity from 0 to 4, with greater scores indicating more severe depression. A total score of 0–7 indicates normal mood, 8–13 indicates mild depression, 14–18 indicates moderate depression, 19–22 indicates severe depression, and ≥23 indicates very severe depression. The scale has been widely used in MDD literature, with indicated severity associated with measures of psychosocial functioning (14).

### Statistical Analyses

Analyses were conducted with statistical package of the social sciences (SPSS) for Windows, version 24. As the current research was exploratory and employed an existing data set, *post hoc* power analyses were not used (8, 38, 39). Descriptive statistics (*M*s, SDs) for the psychosocial dysfunction in the FAST and social cognition in the ACS are presented in **Supplementary eTable 1**. Mediation analyses were conducted with the “Process” macro for SPSS developed by Hayes (40). The Process macro enables nonparametric testing of the indirect relationship *a* × *b* using a bootstrap test of 5,000 resamples. In comparison to the Sobel test, the nonparametric nature of the bootstrap test enables greater statistical power to detect an indirect effect and was therefore appropriate for the present analyses (41).

**Figure 1:**
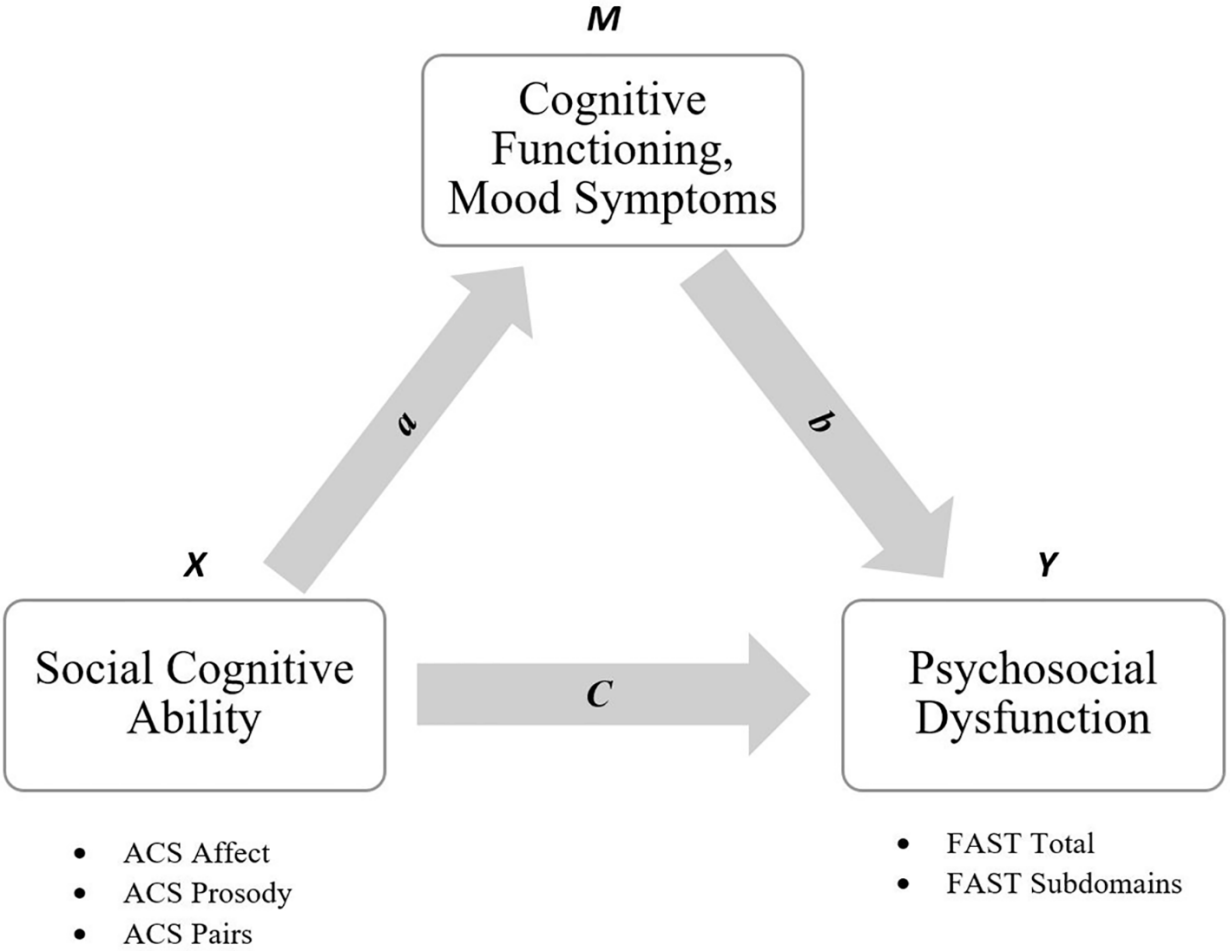
Mediation model for the relationship between social cognitive ability [Advanced clinical solutions (ACS) domains] and psychosocial dysfunction [Functioning Assessment Short Test (FAST) scores], as mediated by cognition and mood symptoms.

Mediation analyses were performed following the procedure recommended by Zhao et al. (41). The notation for this mediation model and the mediation pathways evaluated in the present analyses are presented in **Figure 1**. Following Zhao et al. (41), the only criteria for mediation is a significant indirect effect of *X* (the independent variable) on *Y* (the dependent variable) *via M* (the mediator). The indirect effect is calculated as the product of the coefficients of relationships *a* and *b* (i.e., *a* × *b*). If *a* × *b* is significant, then mediation subtype is determined by the sign (i.e., + or −) of *a* × *b*, and whether the direct effect of *X* on *Y* (i.e., “*c*”) is significant, after adjusting for the removal of variance explained by *a* × *b* (41). Mediation may occur in three patterns: 1) *complementary mediation* if the mediated effect (*a* × *b*) is in the same direction as the direct effect (*c*), 2) *competitive mediation* if *a* × *b* is in the opposite direction to *c*, or 3) *indirect-only mediation* if *a* × *b* is significant while *c* is not significant. In contrast, nonmediation may occur in two patterns: 1) *direct-only nonmediation* occurs if *a* × *b* is not significant while *c* is significant, 2) *no-effect nonmediation* occurs if neither *a* × *b* nor *c* is significant. The total effect (*c*′) indicates the relationship between *X* and *Y*, before adjusting for variance explained by *a* × *b*.

In the current design, a single ACS domain was entered as X, the HAM-D score and composite cognitive domain Z-scores were entered as M, and FAST total score was entered as Y. If the HAM-D score or composite cognition Z-scores produced a significant a × b for a specific ACS domain, then follow-up analyses were conducted with FAST subdomains entered as Y in subsequent models. This exploratory approach enabled testing of domain-specific mediation relationships with each of the three ACS domains, evaluating the potential mechanistic role of cognition and mood symptoms in specific functional deficits. Age, sex, and years of education were entered as covariates, as these factors can influence the relationship between cognition and psychosocial functioning (14, 42).

As the participant sample was composed of currently (n = 42) and previously (n = 69) depressed individuals, it was appropriate to test whether these groups differed in their degree of psychosocial dysfunction (i.e., FAST total score). After adjusting for age, sex, and years of education with an analysis of covariance (ANCOVA), currently depressed participants demonstrated greater overall psychosocial dysfunction (M = 31.18, SD = 17.38) than participants remitted of MDD (M = 15.97, SD = 16.02, p < .001). Following this result, mediation analyses were conducted, which either included or excluded diagnostic status (current MDD, remitted MDD) as a covariate. The inclusion of diagnostic status as a covariate did not alter the significance or magnitude of relationships detected, and hence results that excluded this covariate are presented below.

The present analyses were exploratory in nature, and as such were uncorrected for multiple comparisons (43). Following this approach, any significant results were interpreted as reflecting a narrow (i.e., domain specific) and speculative statistical relationship subject to further testing by future confirmatory analyses (43).

## Results

Initial analyses with ACS affect as *X* and FAST total score as *Y* (see **Figure 1**) indicated no significant indirect relationships when the HAM-D score or composite cognition *Z*-scores were entered as mediators (all 95% CI’s overlap 0). In addition, the direct effect between ACS affect and FAST total score was nonsignificant (all 95% CI’s overlap 0). **Table 2** presents coefficient values, confidence intervals, and mediation status for the analysis of ACS affect.

**Table 2:** Mediation criteria for the ACS Affect–FAST total relationship as mediated by Hamilton Depression Rating Scale (HAM-D) score and composite cognitive domains (*N* = 111).

Potential mediators	Mediation pathway				Mediation type
	*a*	*b*	Indirect effect value and 95% CI	Total (c′) and direct (c) effect value and 95% CI	
HAM-D Score	−.04	1.38**	−.05 [−.99, .97]	*c*′ = .17 [−1.09, 1.42] *c* = .15 [−.81, 1.10]	No effect (nomediation)
Executive Functioning	−.09*	−1.72	.16 [−.06, .41]	*c*′ = .17 [−1.09, 1.42] *c* = .15 [−.81, 1.10]	No effect (nonmediation)
Attention	.11*	.36	.04 [−.16, .30]	*c*′ = .17 [−1.09, 1.42] *c* = .15 [−.81, 1.10]	No effect (nonmediation)
Immediate Memory	.07	−1.90	.14 [−.54, .07]	*c*′ = .17 [−1.09, 1.42] *c* = .15 [−.81, 1.10]	No effect (nonmediation)
Delayed Memory	.12**	1.05	.12 [−.20, .47]	*c*′ = .17 [−1.09, 1.42] *c* = .15 [−.81, 1.10]	No effect (nonmediation)
Semantic Fluency	.07	−1.83	.13 [−.34, .02]	*c*′ = .17 [−1.09, 1.42] *c* = .15 [−.81, 1.10]	No effect (nonmediation)
Spatial Cognition	.02	.15	.01 [−1.01, .97]	*c*′ = .17 [−1.09, 1.42] *c* = .15 [−.81, 1.10]	No effect (nonmediation)

Model adjusted for gender and years of education.

c′ = Relationship between IV, Independent Variable; DV, Dependent Variable; unadjusted for other variables.

c = Relationship between IV and DV, after subtracting variance explained by the indirect effect a × b.

When ACS Prosody was entered as *X* and FAST total score as *Y*, no significant indirect relationships were detected with HAM-D score or composite cognitive domains entered as *M* (all 95% CI’s overlap 0). The direct effect between ACS Prosody and FAST total score was not significant (all 95% CI’s overlap 0). Statistics for the ACS Pairs analysis are presented in **Table 3**.

**Table 3:** Mediation criteria for the ACS Prosody–FAST total relationship as mediated by HAM-D score and composite cognitive domains (*N* = 111).

Potential mediators	Mediation pathway				Mediation type
	a	b	Indirect effect value and 95% CI	Total (c′) and direct (c) effect value and 95% CI	
HAM-D Score	−.72	1.36**	−.97 [−1.96, .04]	*c*′ = −1.85 [−3.38, −.31]* *c* = −.87 [−2.03, .28]	No effect (nonmediation)
Executive Functioning	−.09	−2.32*	.21 [−.13, .53]	*c*′ = −1.85 [−3.38, −.31]* *c* = −.87 [−2.03, .28]	No effect (nonmediation)
Attention	.09	.37	.04 [−.16, .26]	*c*′ = −1.85 [−3.38, −.31]* *c* = −.87 [−2.03,. 28]	No effect (nonmediation)
Immediate Memory	.15**	−1.51	−.21 [−.91, .15]	*c*′ = −1.85 [−3.38, −.31]* *c* = −.87 [−2.03, .28]	No effect (nonmediation)
Delayed Memory	.10	1.22	.12 [−.05, .92]	*c*′ = −1.85 [−3.38, −.31]* *c* = −.87 [−2.03, .28]	No effect (nonmediation)
Semantic Fluency	.08	−1.63	−.14 [−.39, .04]	*c*′ = −1.85 [−3.38, −.31]* *c* = −.87 [−2.03, .28]	No effect (nonmediation)
Spatial Cognition	−.01	−.03	.01 [−.14, .17]	*c*′ = −1.85 [−3.38, −.31]* *c* = −.87 [−2.03, .28]	No effect (nonmediation)

Model adjusted for gender and years of education.

c′ = Relationship between IV and DV, unadjusted for other variables.

c = Relationship between IV and DV, after subtracting variance explained by the indirect effect a × b.

Including ACS Pairs as *X* and FAST total score as *Y* demonstrated a significant indirect effect with executive function (*a* × *b* = .08, CI = .01, .19). As the direct effect was not significant (*c* = −.01, CI = −.49, .48), the pattern of mediation was indirect only. In contrast to executive function, neither HAM-D score nor any other composite cognitive domains resulted in significant indirect effects (all 95% CI’s overlap 0). Statistics for the analysis of ACS Pairs with FAST total score are presented in **Table 4**.

**Table 4:** Mediation criteria for the ACS Pairs–FAST total relationship as mediated by HAM-D score and composite cognitive domains (*N* = 111).

Potential mediators	Mediation pathway				Mediation type
	Coefficients		Indirect effect value and 95% CI	Total (c′) and direct (c) effect value and 95% CI	
	a	b			
HAM-D Score	−.20	1.34**	−.28 [−.78, .19]	*c*′ = −.35 [−.91, .21] *c* = −.01 [−.49, .48]	No effect (nonmediation)
Executive Functioning	−.04*	−2.09	.08 [.01, .19]*	*c*′ = −.35 [−.91, .21] *c* = −.01 [−.49, .48]	Indirect only (mediation)
Attention	.06**	.32	.02 [−.11, .17]	*c*′ = −.35 [−.91, .21] *c* = −.01 [−.49, .48]	No effect (nonmediation)
Immediate Memory	.05**	−1.90	−.09 [−.31, .04]	*c*′ = −.35 [−.91, .21] *c* = −.01 [−.49, .48]	No effect (nonmediation)
Delayed Memory	.06**	1.20	.07 [−.06, .30]	*c*′ = −.35 [−.91, .21] *c* = −.01 [−.49, .48]	No effect (nonmediation)
Semantic Fluency	.08**	−1.79	−.14 [−.35, .03]	*c*′ = −.35 [−.91, .21] *c* = −.01 [−.49, .48]	No effect (nonmediation)
Spatial Cognition	−.01	.18	−.01 [−.05, .06]	*c*′ = −.35 [−.91, .21] *c* = −.01 [−.49, .48]	No effect (nonmediation)

Model adjusted for gender and years of education.

c′ = Relationship between IV and DV, unadjusted for other variables.

c = Relationship between IV and DV, after subtracting variance explained by the indirect effect a × b.

**Figure 2:**
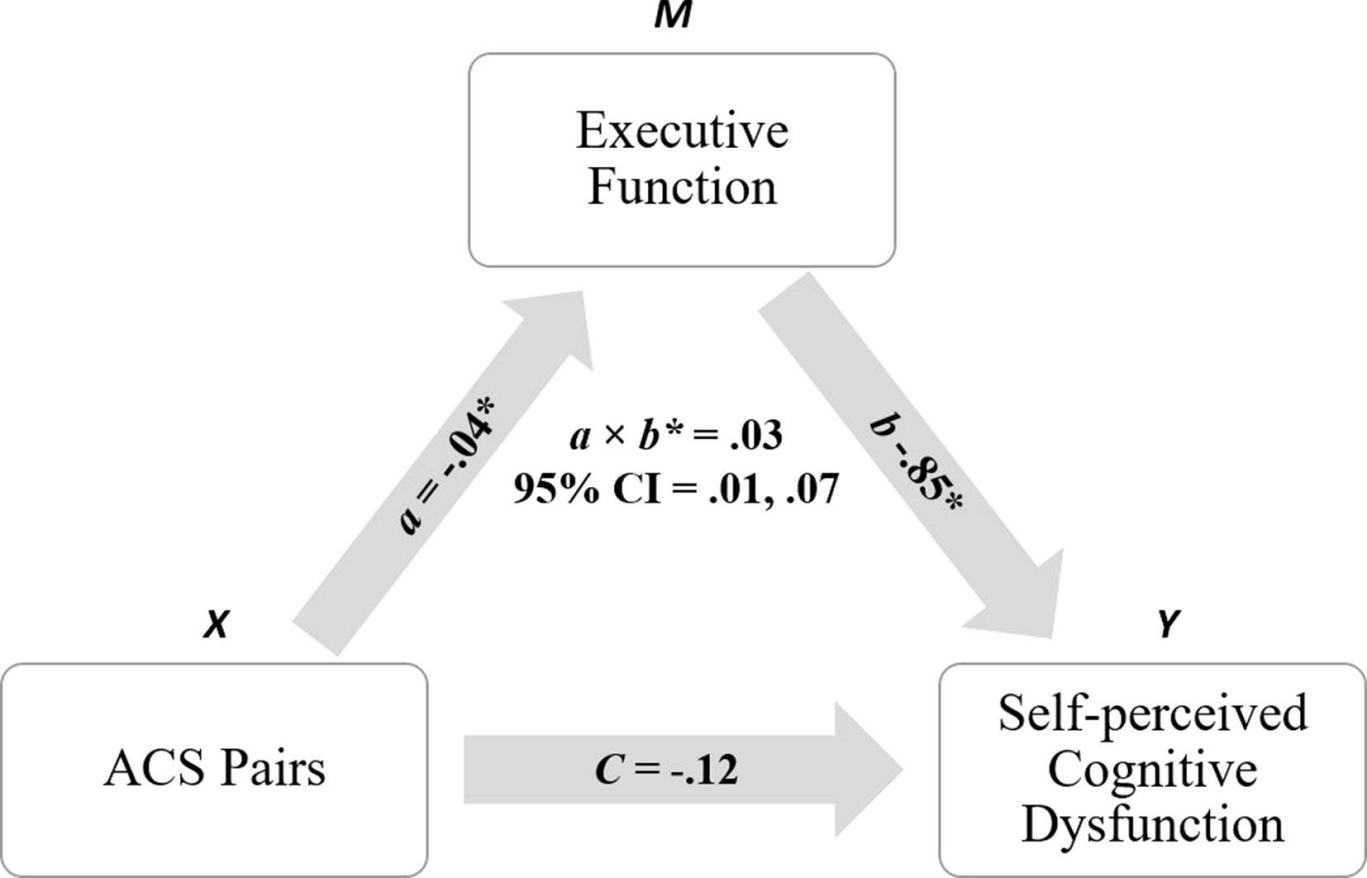
Indirect relationship observed between ACS Pairs (meaning interpretation) and self-perceived cognitive dysfunction, as mediated by executive function. *denotes significance at *p* ≤ .05.

To further analyze the significant indirect effect of ACS Pairs and executive function on FAST total score, follow-up analyses were conducted with FAST subdomains entered as *Y*. A significant indirect effect was demonstrated with subjective cognitive deficits entered as *Y* (*a* × *b* = .03, CI = .01, .07), with the direct effect of ACS Pairs on subjective cognition remaining nonsignificant (*c* = −.12, CI = −.26, .02), resulting in an indirect-only mediation pattern. This mediation relationship is depicted in **Figure 2**. The indirect effect was not significant when any of the remaining FAST subdomains were entered as *Y* (see **Table 5**).

**Table 5:** Mediation criteria for the ACS Pairs–FAST subdomain relationship as mediated by executive function (*N* = 111).

Potential mediators	Mediation pathway				Mediation type
	Coefficients		Indirect effect value and 95% CI	Total (c′) and direct (c) effect value and 95% CI	
	a	b			
Autonomy	−.04*	−.61	.02 [.01, .6]	*c*′ = −.08 [−.20, .03] *c* = −.11 [−.22, .01]	No effect (nonmediation)
Occupational Functioning	−.04*	−.39	.01 [−.04, .08]	*c*′ = −.01 [−.17, .15] *c* = −.02 [−.18, .14]	No effect (nonmediation)
Subjective Cognition	−.04*	−.85*	.03 [.01, .07]*	*c*′ = −.09 [−.23, .05] *c* = −.12 [−.26, .02]	Indirect only (mediation)
Leisure Time	−.04*	−.33	.01 [−.01, .03]	*c*′ = −.04 [−.11, .03] *c* = −.05 [−.12, .01]	No effect (nonmediation)
Financial Issues	−.04	−.24	.01 [−.01, .03]	*c*′ = −.01 [−.06, .04] *c* = −.02 [−.07, .03]	No effect (nonmediation)
Interpersonal Relationships	−.04*	−.14	.01 [−.04, .06]	*c*′ = −.12 [−.31, .06] *c* = −.13 [−.31, .06]	No effect (nonmediation)

Model adjusted for gender and years of education.

c′ = Relationship between IV and DV, unadjusted for other variables.

c = Relationship between IV and DV, after subtracting variance explained by the indirect effect a × b.

*significant at .05; **significant at <.001.

## Discussion

The current study investigated whether the relationship between social cognitive deficits and overall psychosocial dysfunction in lifetime MDD patients is mediated by symptom severity or cognitive function. Our analyses indicated an indirect relationship between meaning interpretation (i.e., theory of mind) and total psychosocial dysfunction, as mediated by executive functioning. Follow-up analyses revealed that executive functioning mediated an indirect relationship between meaning interpretation and self-perceived cognitive dysfunction. While a direct relationship was found between poor prosody interpretation and impaired overall psychosocial functioning, neither HAM-D nor cognitive performance mediated this relationship. Finally, facial affect recognition was not significantly associated with psychosocial functioning either directly or indirectly *via* symptom severity or cognitive function. These results are the first to demonstrate the interaction between social cognition, mood symptoms, and cognitive deficits in their effect on psychosocial dysfunction.

While our previous research on this topic established the link between poor prosody interpretation and psychosocial dysfunction (22), a significant limitation to this research was the possible conflation of reduced social cognition with greater symptom severity and cognitive deficits, as these symptoms typically co-occur. This limitation restricted the conclusion that prosody interpretation was related to psychosocial function directly, as mood symptoms or cognitive deficits could explain this relationship. The present results provide clarity on this point, as the association between prosody interpretation and psychosocial dysfunction was not mediated by mood symptoms or cognitive deficits. In addition, our previous research showed no links between facial affect detection and meaning interpretation with psychosocial dysfunction. However, it remained possible that these social cognitive abilities were associated with psychosocial function indirectly *via* mood symptoms or cognitive deficits. The present results found some evidence for this notion in meaning interpretation, which was indirectly related to psychosocial dysfunction *via* executive function.

Indirect mediation suggests that meaning interpretation does not exert an effect on psychosocial function directly, but rather that meaning interpretation is related to executive function, which in turn is related to perceived cognitive performance. This finding supports the notion that depressed patients’ ability to read the emotions and intentions of others is reliant on executive function, which in turn affects daily functioning. The fact that mediation was only shown for executive function, but not for any other cognitive domains, suggests that greater cognitive complexity inherent to executive functions (e.g., cognitive flexibility, mental updating) is more crucial to theory of mind and psychosocial status than other cognitive functions (e.g., immediate memory). Importantly, follow-up analyses with psychosocial subdomains indicated that executive function was indirectly related to subjective cognitive deficits, but unrelated to other psychosocial subdomains (e.g., autonomy, executive function). The demonstrated link with subjective cognitive deficits suggests that reduced executive function in MDD patients may explain both poor theory of mind and reduced self-perception of cognitive ability. It follows that treating executive deficits may be crucial to attenuating the negative effect of interpersonal difficulties on self-perceived cognitive ability.

The finding that symptom severity did not mediate any relationship between social cognitive abilities and psychosocial function was unexpected. Previous literature has identified that social cognitive deficits and psychosocial dysfunction are elevated in more severely depressed patients (14, 23, 26, 44). Accordingly, it was plausible that greater symptom severity may be the primary mechanism of both social cognitive and psychosocial deficits or, alternatively, may be a significant mechanism by which poor social cognition leads to psychosocial deficits. The present findings provide evidence against this notion, as mood symptoms and cognitive deficits do not mechanistically explain the role of prosody interpretation in reduced psychosocial function. It follows that attenuating mood symptoms is likely insufficient to reduce functional deficits associated with prosody interpretation. Our results underscore the need to develop social cognitive treatments targeting prosody interpretation (27), as current treatments for social cognition in MDD are highly limited (28). In addition, it may be important to incorporate measures of social cognition in clinical assessment/screening procedures for individuals with major depression. At present, no social cognitive screening tools are specialized for depressed patients; however, our results suggest that the ACS battery may serve for this purpose until such an instrument is developed.

It should be acknowledged that the results demonstrated in this study are modest in size and likely reflect subtle relationships between social cognition and functioning. These results confer with previous studies, suggesting social cognitive deficits are more nuanced in MDD by comparison with other psychiatric illnesses (8). In addition, the cross-sectional nature of our results does not enable conclusions regarding the longitudinal association of social cognition with psychosocial function. Also of note is our primarily Australian-Caucasian cultural–ethnic sample, as the present conclusions may not generalize to other social cohorts. In particular, the relationship between prosody and psychosocial functioning may be different in cultures with different languages or social-perceptive features (e.g., Asian or African). It is possible, for instance, that certain cultures place greater emphasis on facial recognition/body language relative to prosody. Finally, our analyses grouped together currently and previously depressed individuals, and our conclusions thus refer to persons with a lifetime occurrence of MDD. Further validation of the present results is warranted in currently depressed and remitted groups specifically, as well as in other cultural samples.

While existing research had identified links between social cognition and psychosocial function in MDD (22, 28, 45), it was previously unclear whether this relationship was conflated by mood symptoms or nonsocial cognitive function. The present findings provide some clarity in this domain, as symptom severity and cognitive deficits did not explain the relationship between social cognitive abilities and psychosocial dysfunction. The exception was executive function, which indirectly mediated a relationship between theory of mind ability and self-perceived cognitive dysfunction, highlighting the importance of treating executive deficits. Taken together, the present results suggest that social cognitive deficits are a subtle yet distinct feature of MDD, which may contribute independently to the pathology of psychosocial dysfunction.

## Ethics Statement

This study was carried out in accordance with the recommendations of the National Statement on Ethical Conduct in Human Research, Human Research Ethics Committee, with written informed consent from all subjects. All subjects gave written informed consent in accordance with the Declaration of Helsinki. The protocol was approved by the Royal Adelaide Hospital Human Research Ethics Committee.

## Author Contributions

BB conceived, designed, coordinated, and supervised the CoFaM-S, from which the current results were derived. MK and BB mutually developed the hypotheses and conceptual outline of the present manuscript. MK analyzed data and wrote the manuscript, while BB edited and provided feedback during the write-up.

## Funding

The work was supported by an unrestricted grant from the James and Diana Ramsay Foundation, Adelaide, Australia. The funding body had no impact on the design or the content of the presented work.

## Conflict of Interest Statement

BB received speaker/consultation fees from AstraZeneca, Lundbeck, Pfizer, Takeda, Servier, Bristol Myers Squibb, Otsuka, and Janssen-Cilag. The remaining author declares that the research was conducted in the absence of any commercial or financial relationships that could be construed as a potential conflict of interest.

## References

[1] Anselmetti S Bechi M Bosia M Quarticelli C Ermoli E Smeraldi E ‘Theory’ of mind impairment in patients affected by schizophrenia and in their parents. Schizophr Res (2009) 115(2):278–85. 10.1016/j.schres.2009.09.018 19818586

[2] Frith CD Corcoran R Exploring ‘theory of mind’ in people with schizophrenia. Psychol Med (1996) 26(3):521–30. 10.1017/S0033291700035601 8733211

[3] Persad SM Polivy J Differences between depressed and nondepressed individuals in the recognition of and response to facial emotional cues. J Abnorm Psychol (1993) 102(3):358. 10.1037//0021-843X.102.3.358 8408947

[4] LeMoult J Joormann J Sherdell L Wright Y Gotlib IH Identification of emotional facial expressions following recovery from depression. J Abnorm Psychol (2009) 118(4):828. 10.1037/a0016944 19899852 PMC2837802

[5] Air T Weightman MJ Baune BT Symptom severity of depressive symptoms impacts on social cognition performance in current but not remitted major depressive disorder. Front Psychol (2015) 6:1118. 10.3389/fpsyg.2015.01118 26300814 PMC4523699

[6] Demenescu LR Kortekaas R Boer JA Aleman A Impaired attribution of emotion to facial expressions in anxiety and major depression. PloS one (2010) 5(12):e15058. 10.1371/journal.pone.0015058 21152015 PMC2995734

[7] Hasselbalch BJ Knorr U Kessing LV Cognitive impairment in the remitted state of unipolar depressive disorder: a systematic review. J Affective Disord (2011) 134(1–3):20–31. 10.1016/j.jad.2010.11.011 21163534

[8] Weightman MJ Air TM Baune BT A review of the role of social cognition in major depressive disorder. Front Psychiatry (2014) 5:179. 10.3389/fpsyt.2014.00179 25566100 PMC4263091

[9] Brenner HD Hodel B Roder V Corrigan P Treatment of cognitive dysfunctions and behavioral deficits in schizophrenia. Schizophr Bull (1992) 18(1):21. 10.1093/schbul/18.1.21 1553495

[10] Williams LM Whitford TJ Flynn G Wong W Liddell BJ Silverstein S General and social cognition in first episode schizophrenia: identification of separable factors and prediction of functional outcome using the IntegNeuro test battery. Schizophr Res (2008) 99(1):182–91. 10.1016/j.schres.2007.10.019 18053688

[11] Dhar M Been PH Minderaa RB Althaus M Information processing differences and similarities in adults with dyslexia and adults with attention deficit hyperactivity disorder during a continuous performance test: a study of cortical potentials. Neuropsychologia (2010) 48(10):3045–56. 10.1016/j.neuropsychologia.2010.06.014 20600194

[12] Harvey PD Penn D Social cognition: the key factor predicting social outcome in people with schizophrenia? Psychiatry (Edgmont) (2010) 7(2):41. PMC2848464 20376275

[13] Baune BT Miller R McAfoose J Johnson M Quirk F Mitchell D The role of cognitive impairment in general functioning in major depression. Psychiatry Res (2010) 176(2–3):183–9. 10.1016/j.psychres.2008.12.001 20138370

[14] Evans VC Iverson GL Yatham LN Lam RW The relationship between neurocognitive and psychosocial functioning in major depressive disorder: a systematic review. J Clin Psychiatry (2014) 75(12):1359–70. 10.4088/JCP.13r08939 25551235

[15] Gotlib IH Joormann J Cognition and depression: current status and future directions. Ann Rev Clin Psychol (2010) 6:285–312. 10.1146/annurev.clinpsy.121208.131305 20192795 PMC2845726

[16] Knight MJ Baune BT Executive subdomains are differentially associated with psychosocial outcomes in major depressive disorder. Front Psychiatry (2018a) 9:309. 10.3389/fpsyt.2018.00309 30042703 PMC6048277

[17] McDermott LM Ebmeier KP A meta-analysis of depression severity and cognitive function. J Affective Disord (2009) 119(1):1–8. 10.1016/j.jad.2009.04.022 19428120

[18] McIntyre RS Soczynska JZ Woldeyohannes HO Alsuwaidan MT Cha DS Carvalho AF The impact of cognitive impairment on perceived workforce performance: results from the International Mood Disorders Collaborative Project. Compr psychiatry (2015) 56:279–82. 10.1016/j.comppsych.2014.08.051 25439523

[19] Baune BT Air T Clinical, functional, and biological correlates of cognitive dimensions in major depressive disorder—rationale, design, and characteristics of the Cognitive Function and Mood Study (CoFaM-Study). Front Psychiatry (2016) 7:150. 10.3389/fpsyt.2016.00150 27616997 PMC4999943

[20] Knight MJ Air T Baune BT The role of cognitive impairment in psychosocial functioning in remitted depression. J Affective Disord (2018) 235:129–34. 10.1016/j.jad.2018.04.051 29655074

[21] Xiang X An R The impact of cognitive impairment and comorbid depression on disability, health care utilization, and costs. Psychiatric Serv (2015) 66(11):1245–8. 10.1176/appi.ps.201400511 26073413

[22] Knight MJ Baune BT Social cognitive abilities predict psychosocial dysfunction in major depressive disorder. Depress Anxiety (2018b) 36:1–9. 10.1002/da.22844 30211966

[23] Gollan JK McCloskey M Hoxha D Coccaro EF How do depressed and healthy adults interpret nuanced facial expressions? J Abnorm Psychol (2010) 119(4):804. 10.1037/a0020234 20939654 PMC3805828

[24] Johnson TJ DiLorenzo TM Social information processing biases in depressed and nondepressed college students. J Social Behav Pers (1998) 13(3):517.

[25] Bhagwagar Z Cowen PJ Goodwin GM Harmer CJ Normalization of enhanced fear recognition by acute SSRI treatment in subjects with a previous history of depression. Am J Psychiatry (2004) 161(1):166–8. 10.1176/appi.ajp.161.1.166 14702268

[26] Judd LL Akiskal HS Zeller PJ Paulus M Leon AC Maser JD Psychosocial disability during the long-term course of unipolar major depressive disorder. Arch Gen Psychiatry (2000) 57(4):375–80. 10.1001/archpsyc.57.4.375 10768699

[27] Knight MJ Baune BT Psychosocial dysfunction in major depressive disorder–rationale, design, and characteristics of the Cognitive and Emotional Recovery Training Program for Depression (CERT-D). Front Psychiatry (2017) 8:280. 10.3389/fpsyt.2017.00280 29312014 PMC5732931

[28] Weightman MJ Knight MJ Baune BT A systematic review of the impact of social cognitive deficits on psychosocial functioning in major depressive disorder and opportunities for therapeutic intervention. Psychiatry Res (2019) 274:195–212. 10.1016/j.psychres.2019.02.035 30807971

[29] American Psychiatric Association DSM IV Diagnostic and statistical manual of mental disorders. Washington, DC: American Psychiatric Association (1994).

[30] Sheehan D Lecrubier Y Sheehan KH Sheehan K Amorim P Janavs J Diagnostic psychiatric interview for DSM-IV and ICD-10. J Clin Psychiatry (1998) 59:22–33. 9881538

[31] Hamilton M The Hamilton Depression Scale—accelerator or break on antidepressant drug discovery. Psychiatry (1960) 23:56–62. 10.1136/jnnp.23.1.56 24443712

[32] Holdnack J Goldstein G Drozdick L Social perception and WAIS-IV performance in adolescents and adults diagnosed with Asperger’s syndrome and autism. Assessment (2011) 18(2):192–200. 10.1177/1073191110394771 21220381

[33] Kandalaft MR Didehbani N Cullum CM Krawczyk DC Allen TT Tamminga CA The Wechsler ACS social perception subtest: a preliminary comparison with other measures of social cognition. J Psychoeduc Assess (2012) 30(5):455–65. 10.1177/0734282912436411

[34] Randolph C Tierney MC Mohr E Chase TN The Repeatable Battery for the Assessment of Neuropsychological Status (RBANS): preliminary clinical validity. J Clin Exp Neuropsychol (1998) 20(3):310–9. 10.1076/jcen.20.3.310.823 9845158

[35] Davis H Keller F Colorado assessment tests (CATS), version 1.2. Colorado Springs (CO): Colorado Assessment Tests (2002).

[36] Mueller ST Piper BJ The psychology experiment building language (PEBL) and PEBL test battery. J Neurosci Methods (2014) 222:250–9. 10.1016/j.jneumeth.2013.10.024 PMC3897935 24269254

[37] Godard J Baruch P Grondin S Lafleur MF Psychosocial and neurocognitive functioning in unipolar and bipolar depression: a 12-month prospective study. Psychiatry Res (2012) 196(1):145–53. 10.1016/j.psychres.2011.09.013 22370154

[38] Goodman SN Berlin JA The use of predicted confidence intervals when planning experiments and the misuse of power when interpreting results. Ann Int Med (1994) 121(3):200–6. 10.7326/0003-4819-121-3-199408010-00008 8017747

[39] O’Keefe DJ Brief report: *post hoc* power, observed power, *a priori* power, retrospective power, prospective power, achieved power: sorting out appropriate uses of statistical power analyses. Commun Methods Meas (2007) 1(4):291–9. 10.1080/19312450701641375

[40] Hayes AF PROCESS: a versatile computational tool for observed variable mediation, moderation, and conditional process modeling. University of Kansas, KS (2012). http://www.afhayes.com/public/process2012.pdf

[41] Zhao X Lynch JG Jr. Chen Q Reconsidering Baron and Kenny: myths and truths about mediation analysis. J Consum Res (2010) 37(2):197–206. 10.1086/651257

[42] Mackin RS Areán PA Impaired financial capacity in late life depression is associated with cognitive performance on measures of executive functioning and attention. J Int Neuropsychol Soc (2009) 15(5):793–8. 10.1017/S1355617709990300 19635176

[43] Goeman JJ Solari A Multiple testing for exploratory research. Stat Sci (2011) 26(4):584–97. 10.1214/11-STS356

[44] Cambridge OR Knight MJ Mills N Baune BT The clinical relationship between cognitive impairment and psychosocial functioning in major depressive disorder: a systematic review. Psychiatry Res (2018) 269:157–71. 10.1016/j.psychres.2018.08.033 30149273

[45] Szanto K Dombrovski AY Sahakian BJ Mulsant BH Houck PR Reynolds CF Social emotion recognition, social functioning, and attempted suicide in late-life depression. Am J Geriatric Psychiatry (2012) 20(3):257–65. 10.1097/JGP.0b013e31820eea0c PMC3286029 22354116

