# Supplementary material for: The Direct and Indirect Relationship Between Social Cognition and Psychosocial Dysfunction in Major Depressive Disorder
Source: Front Psychiatry. 2019 May 17;10:347. doi: 10.3389/fpsyt.2019.00347 (PMC6533355; doi:10.3389/fpsyt.2019.00347)
Supplement: Supplementary file 1 [file Table_1.doc]

| **Supplementary eTable 1.** | | | | | | | | |  | |  |  |
| --- | --- | --- | --- | --- | --- | --- | --- | --- | --- | --- | --- | --- |
| *Mean FAST score, FAST subdomain score, and performance in ACS tests across the participant sample (N = 111). Standard deviations are shown in parentheses.* | | | | | | | | | | | | |
| FAST Subdomains | | | | | | | | | ACS tests | | | |
| FAST total Score | Autonomy | Occupational Functioning | Subjective Cognition | Leisure Time | Financial Issues | Interpersonal relationships | ACS Affect | ACS Prosody | | ACS Pairs | | |
| 19.23 (12.80) | 2.55 (2.70) | 5.40 (3.92) | 4.03 (3.45) | 1.44  (1.55) | .73 (1.3) | 4.83 (4.27) | 18.96 (3.45) | 8.87 (1.84) | | 30.16 (5.02) | | |
